# Over-the-scope clip hemostasis assisted by resuscitative endovascular balloon occlusion of the aorta for life-threatening duodenal ulcer bleeding

**DOI:** 10.1055/a-2886-4526

**Published:** 2026-06-19

**Authors:** Chikamasa Ichita, Miki Nagayama, Tetsuya Hattori

**Affiliations:** 1 Department of Gastroenterology 38088 Kitasato University School of MedicineSagamiharaJapan; 2 Gastroenterology Medicine Center 13619 Shonan Kamakura General HospitalKamakuraJapan

Resuscitative endovascular balloon occlusion of the aorta (REBOA) temporarily controls hemorrhage by inflating an aortic balloon inserted via femoral arterial access ( Fig. 1 ). It has been used mainly in trauma and obstetric hemorrhage, 1​ ​2​ whereas reports in gastrointestinal bleeding remain limited. 3​ In nonvariceal gastrointestinal bleeding, Zone I occlusion can reduce splanchnic arterial inflow; however, complete occlusion should be minimized, optimally to < 30 minutes because of distal ischemia and related adverse effects, including ischemia–reperfusion injury, renal impairment, bowel or lower-limb ischemia, and access-site vascular complications, 4​ ( Fig. 2 ). Rapid mechanical hemostasis with an over-the-scope clip (OTSC) may therefore be useful. 5​

**Fig. 1:**
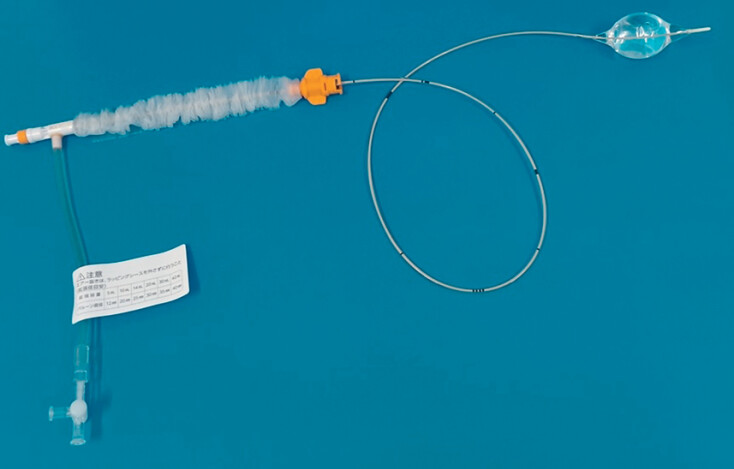
A photograph of the REBOA balloon catheter used in this case.

**Fig. 2:**
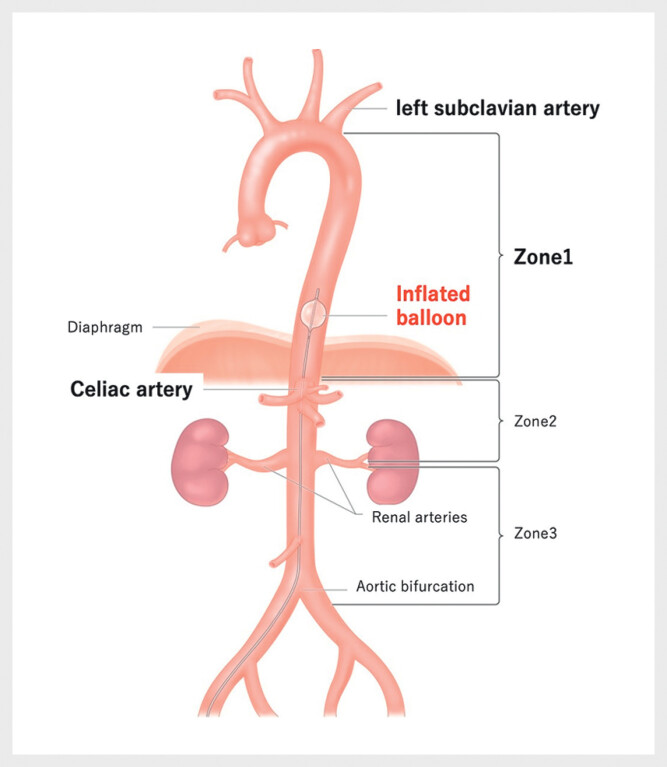
Schematic illustration of REBOA zones. Zone I extends from the origin of the left subclavian artery to the celiac artery, and the balloon is positioned in Zone I.

A man in his 50 s with alcohol-related cirrhosis developed cardiac arrest after hematemesis and was resuscitated. Contrast-enhanced computed tomography showed extravasation in the descending duodenum ( Fig. 3 ). On arrival, he had hemorrhagic shock despite intubation and vasopressors. As surgery and interventional radiology were not immediately available, a REBOA catheter was inserted via the left femoral artery and positioned in Zone I, with the balloon deflated, before emergency endoscopy.

**Fig. 3:**
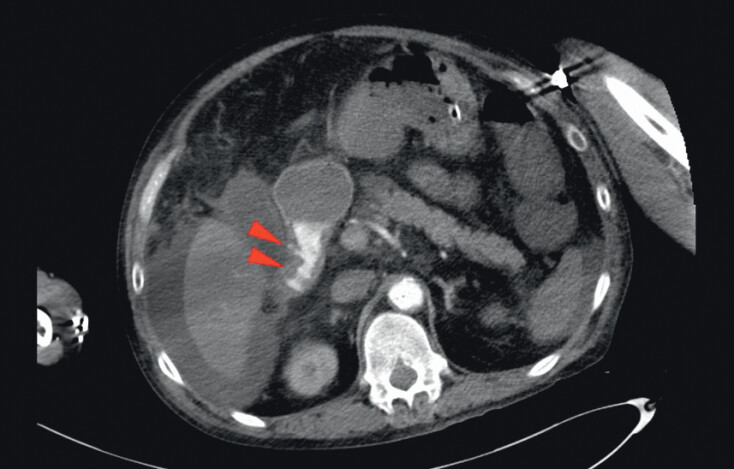
Contrast-enhanced computed tomography obtained at the referring hospital. Active contrast extravasation was observed in the descending part of the duodenum (red arrowheads).

Video 1 demonstrates the REBOA-assisted hemostatic strategy. First, balloon inflation was delayed until massive duodenal bleeding was confirmed and the endoscope had reached the presumed source. Inflation reduced bleeding and revealed a deep ulcer on the inferior wall of the superior duodenal angle, but the exact bleeding point was obscured under complete occlusion. Second, brief titrated deflation to partial aortic occlusion under hemodynamic and endoscopic monitoring revealed focal active bleeding from the right side of the ulcer; a marking clip was placed and the balloon was reinflated. Third, rapid mechanical hemostasis was achieved by deploying an OTSC using the marking clip as a landmark. Final deflation showed no rebleeding, bridging the patient to definitive coil embolization ( Fig. 4 ). No subsequent rebleeding or renal impairment occurred.

**Video 1** Over-the-scope clip hemostasis assisted by resuscitative endovascular balloon occlusion of the aorta for life-threatening duodenal ulcer bleeding, highlighting delayed inflation, brief planned deflation, and rapid mechanical hemostasis.


**Fig. 4:**
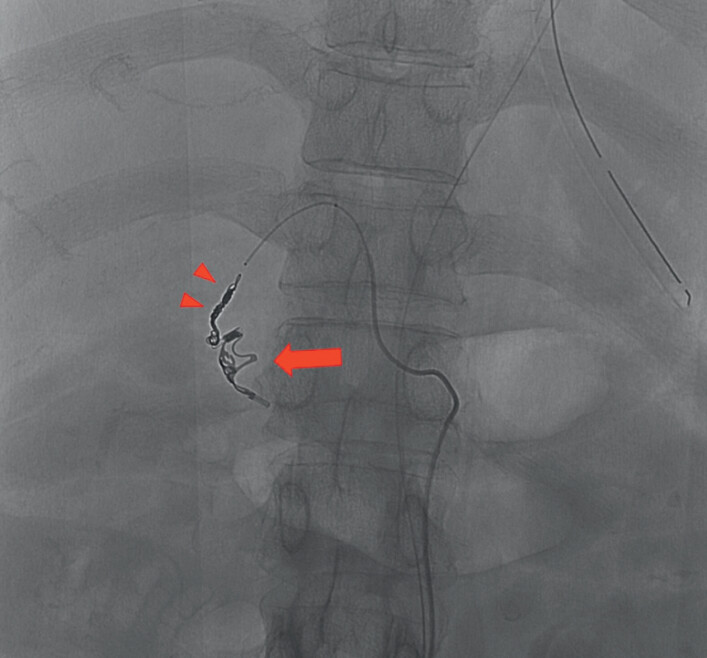
An angiographic image after definitive transcatheter arterial embolization. The red arrow indicates the OTSC, and the red arrowheads indicate the embolization coils.

Endoscopy_UCTN_Code_TTT_1AO_2AD

## References

[1] DuBose J J Scalea T M Brenner M The AAST prospective Aortic Occlusion for Resuscitation in Trauma and Acute Care Surgery registry: data on contemporary utilization and outcomes of aortic occlusion and resuscitative balloon occlusion of the aorta J Trauma Acute Care Surg 2016 81 409 419 27050883 10.1097/TA.0000000000001079

[2] Kamijo K Nakajima M Shigemi D Resuscitative endovascular balloon occlusion of the aorta for life-threatening postpartum hemorrhage: a nationwide observational study in Japan J Trauma Acute Care Surg 2022 93 418 423 35444149 10.1097/TA.0000000000003650

[3] Sano H Tsurukiri J Hoshiai A Resuscitative endovascular balloon occlusion of the aorta for uncontrollable nonvariceal upper gastrointestinal bleeding World J Emerg Surg 2016 11 20 27213011 10.1186/s13017-016-0076-3 PMC4875689

[4] Joint Trauma System. Resuscitative endovascular balloon occlusion of the aorta (REBOA) for hemorrhagic shock. Clinical Practice Guideline ID 38. 2025. https://jts.health.mil/assets/docs/cpgs/REBOA_for_Hemorrhagic_Shock_4.3.2026_ID38_v1.4.pdf. cited May 14, 2026

[5] Schmidt A Gölder S Goetz M Over-the-scope clips are more effective than standard endoscopic therapy for patients with recurrent bleeding of peptic ulcers Gastroenterology 2018 155 674 6.86E8 29803838 10.1053/j.gastro.2018.05.037

